# Periostin^+^cancer‐associated fibroblasts promote lymph node metastasis by impairing the lymphatic endothelial barriers in cervical squamous cell carcinoma

**DOI:** 10.1002/1878-0261.12837

**Published:** 2020-11-12

**Authors:** Wen‐Fei Wei, Xiao‐Jing Chen, Luo‐Jiao Liang, Lan Yu, Xiang‐Guang Wu, Chen‐Fei Zhou, Zi‐Ci Wang, Liang‐Sheng Fan, Zheng Hu, Li Liang, Wei Wang

**Affiliations:** ^1^ Department of Obstetrics and Gynecology the First Affiliated Hospital of Guangzhou Medical University China; ^2^ Department of Gynecological Oncology the First Affiliated Hospital Sun Yat‐sen University Guangzhou China; ^3^ Precision Medicine Institute the First Affiliated Hospital Sun Yat‐sen University Guangzhou China; ^4^ Department of Pathology Nanfang Hospital Southern Medical University Guangzhou China

**Keywords:** cancer‐associated fibroblasts, cervical squamous cell carcinoma, lymph node metastasis, lymphatic endothelial barrier, periostin

## Abstract

Lymph node metastasis (LNM), a critical prognostic determinant in cancer patients, is critically influenced by the presence of numerous heterogeneous cancer‐associated fibroblasts (CAFs) in the tumor microenvironment. However, the phenotypes and characteristics of the various pro‐metastatic CAF subsets in cervical squamous cell carcinoma (CSCC) remain unknown. Here, we describe a CAF subpopulation with elevated periostin expression (periostin^+^CAFs), located in the primary tumor sites and metastatic lymph nodes, that positively correlated with LNM and poor survival in CSCC patients. Mechanistically, periostin^+^CAFs impaired lymphatic endothelial barriers by activating the integrin‐FAK/Src‐VE‐cadherin signaling pathway in lymphatic endothelial cells and consequently enhanced metastatic dissemination. In contrast, inhibition of the FAK/Src signaling pathway alleviated periostin‐induced lymphatic endothelial barrier dysfunction and its related effects. Notably, periostin^‐^CAFs were incapable of impairing endothelial barrier integrity, which may explain the occurrence of CAF‐enriched cases without LNM. In conclusion, we identified a specific periostin^+^CAF subset that promotes LNM in CSCC, mainly by impairing the lymphatic endothelial barriers, thus providing the basis for potential stromal fibroblast‐targeted interventions that block CAF‐dependent metastasis.

## Graphical abstract

In this study, we identified a specific periostin^+^CAF subset that promoted lymph node metastasis (LNM) in cervical squamous cell carcinoma (CSCC). Periostin^+^CAFs impaired lymphatic endothelial barriers by activating the integrin‐FAK/Src‐VE‐cadherin signaling pathway in lymphatic endothelial cells and consequently enhanced metastatic dissemination. Blocking periostin or its downstream effectors (using inhibitors) FAK (PF‐562271)/Src (BMS‐354825 monohydrate) may inhibit periostin^+^CAFs‐induced LNM.

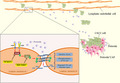

**Abbreviations**
CAFs: cancer‐associated fibroblasts
CM: conditioned medium
CSCC: cervical squamous cell carcinoma
HDLECs: human dermal lymphatic endothelial cells
LN: lymph node
LNM: lymph node metastasis
LVs: lymphatic vessels
MFI: mean fluorescence intensity
NC: negative control
NOFs: normal fibroblasts
SFM: serum‐free media
TAMs: tumor‐associated macrophages
TEM: transmission electron microscopy
TME: tumor microenvironment

## 1 Introduction

Cancer‐associated fibroblasts (CAFs) are activated fibroblasts that constitute the most abundant stromal components in several types of cancers [1, 2]. The heterogeneity of CAFs, that enables them to respond differently to various microenvironmental signals and become functionally opposing factors during cancer progression presents a major challenge in the clinical management of tumor malignancy [2, 3]. Although their distinct cellular origins have been elucidated, the mechanisms underlying CAF‐mediated lymph node metastasis (LNM) are poorly understood [4, 5]. In most cases, the establishment of lymphatic metastases occurs when tumor cells escape from the primary tumor site, invade the lymphatic vessel (LV), survive in the circulation, and subsequently grow at a new site [6]. Previous studies have emphasized that the LVs within the tumor microenvironment (TME) undergo dynamic changes that facilitate metastasis [7]. However, studies focused on analyzing the LV properties affected by the distinct CAF subtypes in various cancer types are limited.

Dynamic changes in the regional lymphatic vessels that facilitate tumor cell dissemination play an important pro‐metastatic role in solid tumors, particularly in cervical squamous cell carcinoma (CSCC) [8, 9]. One of the major barriers of tumor cell intravasation through the lymphatic system is LV integrity [10], that is, strictly related to protein organization in the interendothelial adherens junctions (AJs) [11]. In addition, the maintenance of the lymphatic endothelial barrier integrity and its key function depends on the cells or cytokines within the regional microenvironment [7, 10, 12]. As one of the most abundant stromal cells, fibroblasts have a major physiological function in maintaining the homeostasis and structural integrity of most tissues. This function seizes during metastatic progression to induce the formation of a pro‐metastatic microenvironment. Several studies have revealed that CAFs contribute to the disruption of homeostatic regulation via paracrine signaling and physical interactions, such as control of tissue architecture, adhesion, and proliferation [4, 13]. Paracrine signaling in the TME is essential for CAF phenotyping [14], whereas distinct CAF‐derived signals serve as determinants of site‐specific metastasis in various cancers [15]. These findings suggest that certain CAF subpopulations may have a profound impact on LNM of CSCC. However, it remains unclear whether CAF‐mediated regulation of TME remodeling is associated with impairment of the lymphatic endothelial barriers. Moreover, the phenotypes and characteristics of pro‐metastatic CAF subsets in CSCC, as well as the mechanisms underlying CAF‐mediated lymphatic remodeling in the TME, remain unknown.

In this study, we investigated a specific CAF subset with high expression of periostin (periostin^+^CAFs) that accumulated in the TME and infiltrated the stromal regions surrounding the metastatic lymph nodes (LNs) in CSCC. We discovered that periostin^+^CAFs impaired the lymphatic endothelial barriers by activating the integrin‐FAK/Src‐VE‐cadherin signaling pathway in lymphatic endothelial cells. However, inhibition of FAK/Src signaling alleviated the periostin^+^ CAF‐induced disruption of endothelial barrier integrity. These results highlight the heterogeneity of CAFs in CSCC and help to identify periostin^+^CAFs as novel pro‐metastatic factors that promote LNM by breaching lymphatic integrity. Periostin^+^CAFs may serve as a potential target for future studies focused on the prevention and treatment of CSCC metastasis.

## 2 Materials and methods

### 2.1 Clinical specimens

All cervical specimens were obtained from voluntarily consenting patients at the Department of Gynecological Oncology of The First Affiliated Hospital of Guangzhou Medical University and Nanfang Hospital (Guangzhou, China). This study was approved by the Institutional Research Ethics Committee and compliant with the principles set forth by the Declaration of Helsinki Principles.

The paraffin‐embedded cervical specimens included 57 CSCC cases without LNM and 20 CSCC cases with LNM and were subjected to immunohistochemical and immunofluorescence analyses. The fresh samples were used for fibroblast isolation and included eight normal cervical tissue samples from multiple hysteromyoma patients that underwent hysterectomy and 27 cervical cancer tissue samples from CSCC patients that underwent abdominal radical hysterectomy without prior radiotherapy and chemotherapy. Only 12 out of the 27 cervical cancer cases submitted for pathological examination after surgery were confirmed to contain LNM.

### 2.2 Cell cultures and transfection

The human cervical cancer cell line, SiHa., was purchased from American Type Culture Collection (ATCC, Manassas, VA, USA) and cultured according to the supplier's guidelines. Human dermal lymphatic endothelial cells (HDLECs) were purchased from ScienCell Research Laboratories (Carlsbad, CA, USA) and cultured in endothelial cell medium (ECM; ScienCell) with 5% FBS (Gibco, Invitrogen, Carlsbad, CA, USA). The SiHa cells were transfected with lenti‐mCherry; cells stably expressing mCherry fluorescent protein signals were selected for further experiments. On the other hand, the HDLECs were transfected with lenti‐GFP to stably express GFP fluorescent protein signals.

### 2.3 Isolation and identification of CAFs and NOFs from fresh CSCC samples and normal cervical samples

The fresh cervical tissue samples were washed with Dulbecco's modified Eagle's medium (DMEM; Invitrogen) supplemented with 10% FBS (Gibco) and finely minced into small pieces (approximately 0.2 × 0.2 mm). The minced samples were incubated in fresh culture medium for 24 h to allow attachment to the culture plate. Following incubation, the unattached cells were removed and the remaining cells were allowed to grow on the plate for three to four weeks. During this period, the medium was replenished once every 2 days until the fibroblasts started to grow out. The normal fibroblasts (NOFs) were examined by > 90% positive immunofluorescence staining for vimentin and by negative staining for α‐SMA, FAP, CD31, and pan‐cytokeratin. Vimentin, α‐SMA, and FAP are fibroblast markers, while CD31 and pan‐cytokeratin are endothelial and epithelial cell markers, respectively. The CAFs were examined by > 90% positive staining for α‐SMA, FAP, and vimentin and by negative staining for CD31 and pan‐cytokeratin to ensure no contamination of other cell types before further experiments. The fibroblasts isolated from LN‐positive tumor tissue, LN‐negative tumor tissue, and normal cervical tissue were defined as CAFs^LNM^, CAFs^non‐LNM^, and NOFs, respectively. Primary fibroblasts with no more than 10 passages were used for the experiments [16, 17].

### 2.4 Preparation of conditioned medium

The fibroblasts were grown to ~ 80% confluence in growth culture medium. The cells were washed and incubated in endothelial cell medium (ECM) (ScienCell) with full supplements and 2% FBS (Gibco) at 37 °C for 48 h. The fibroblast‐conditioned medium (CM) was subsequently harvested, centrifuged at 2000 ***g*** for 5 min, and filtered using 0.2‐μm membrane syringe filters to eliminate cell debris. The cleared CM was collected and added to the endothelial cell monolayer for *in vitro* or *in vivo* permeability assays [16, 17].

### 2.5 RNA extraction and qRT–PCR

RNA was extracted from the cells using TRIzol (Invitrogen). qRT–PCR was performed as previously described [18]. The primer sequences are shown in Table S1. The expression level of each mRNA was normalized to that of GAPDH.

### 2.6 Western blotting

Western blotting assay was performed as previously described [18]. The primary antibodies were as follows: anti‐CD31, anti‐pan‐CK, antivimentin, anti‐FAP, anti‐αSMA, anti‐LYVE‐1 antibody, antiperiostin (Abcam, Cambridge, MA, USA), anti‐phospho‐AKT (Ser 473), anti‐AKT, anti‐phospho‐ERK1/2 (T202/Y204), anti‐ERK1/2, anti‐Src, anti‐phospho‐Src (Tyr416), anti‐FAK, anti‐phospho‐FAK (Tyr397), anti‐JNK, anti‐phospho‐JNK, anti‐VE‐cadherin, anti‐VE‐cadherin (phospho Y685), and anti‐GAPDH antibody (CST, Danvers, MA, USA). The secondary antibodies were horseradish peroxidase‐conjugated anti‐rabbit or anti‐mouse immunoglobulin‐G antibody (Abcam).

### 2.7 Human cytokine array

Assessment of cytokines secreted by fibroblasts was performed using human cytokine antibody array (RayBio Human Cytokine antibody array QAH‐CYT9 and AAH‐CYT‐G9) that detects 91 cytokines. Medium from ~ 80% confluent fibroblasts (NOFs‐1, NOFs‐2, NOFs‐3, CAFs^non‐LNM^‐1, CAFs^non‐LNM^‐2, CAFs^LNM^‐1, and CAFs^LNM^‐2, respectively) was replaced with serum‐free medium. Forty‐eight hours later, the media was harvested, and particulates were removed by brief centrifugation, aliquoted, and frozen. Fresh aliquots were quantified, and ~ 100 μL was used for the assay according to the manufacturer's protocol. After the experimental procedure, the slides were scanned with a GenePix 4000B scanner (Axon Instruments, GenePix version 5.0, San Jose, CA, USA) and the signal values were analyzed using the RayBiotech analysis tool, which is based on Microsoft Excel software and specifically designed to analyze the data of Human Cytokine Antibody Array. In this analysis tool, the signals are normalized using internal positive and negative controls included on the array.

### 2.8 ELISA assay

ELISA assay was performed as Li *et al*. described [19]. Briefly, after screening by antibody array, periostin level of CM samples was measured by ELISA (Abcam) according to the manufacturer's instructions. CM samples were coated in the plates for 2.5 h at room temperature. The plates then were incubated with a biotin‐conjugated antibody for 2 h. After washing, HRP‐conjugated streptavidin was added to combine with any biotin catalyzed by the TMB reagent. Finally, sulfuric acid was used to stop the catalytic reaction and the optical density determined via Synergy Neo2 Multi‐Mode Reader (BioTek, Winooski, VT, USA).

### 2.9 Immunohistochemistry

Tissue sections were subjected to immunohistochemical analysis as described previously [20]. The primary antibodies were as follows: antiperiostin (Abcam) and anti‐mCherry antibody (Abcam). The secondary antibodies were horseradish peroxidase‐conjugated anti‐rabbit immunoglobulin‐G antibody (Abcam).

### 2.10 Staining assessment

For staining results, all areas of each sample were examined and that with the greatest immunoreactivity was selected for quantification. The immunoreactivity score of periostin was calculated by summing the score for the percentage of positive cells and the intensity score. The tissue section was scored as the percentage of stained cytoplasm or nuclear in stromal cells (0 points for no cells stained, 1 points for < 25%, 2 points for 25–75%, and 3 points for > 75% of cells stained), and the staining intensity of immunoreactivity was graded on a scale of 0 to 3. The immunoreactivity score (IRS) was resulted from the multiplication of both parameters. Samples were scored as follows: negative (IRS = 0–2) and positive (IRS = 3–9).

### 2.11 Immunofluorescence assay

The HDLECs were grown to confluence on confocal dishes. After pretreatment, the cells were fixed with 4% paraformaldehyde for 30 min and blocked with 5% BSA (Solarbio, Beijing, China) for 30 min. The cells were then incubated with primary antibodies: anti‐VE‐cadherin (Abcam) in a solution containing 0.2% saponin and 1% BSA at 4 °C overnight. The cells were washed, incubated with Alexa Fluor‐conjugated secondary antibodies (Abcam), and mounted in medium containing DAPI (Vector Labs, Burlingame, CA, USA). Images were obtained using a Zeiss LSM 880 with Airyscan (Carl Zeiss Microscopy GmbH, Oberkochen, Germany). The fluorescence intensity of the protein was analyzed using ZEN2.1/ZEN2 software (Carl Zeiss Microscopy GmbH).

Serial paraffin sections (4 µm) from human CSCC tissues were analyzed by immunofluorescence using Opal 4‐Color fluorescent IHC kit (PerkinElmer, Waltham, MA, USA) [21]. After deparaffinization, the sections were microwaved in antigen retrieval buffer for 45 s at 100 °C and then at 10–20% reduced heat for 15 min to prevent boiling. The sections were washed and blocked at room temperature for 10 min, followed by incubation with the primary antibody. The HRP‐conjugated secondary antibody was dropped onto the slides and incubated at room temperature for 10 min; subsequently, the TSA working buffer containing Opal 520, Opal 570, Opal 650, and DAPI was used for signal amplification. After removing the primary and secondary antibodies by microwaving the slides, the same procedures were repeated with the next primary antibody and TSA working buffer. The primary antibodies include anti‐α‐SMA, anti‐LYVE‐1, antiperiostin, and anti‐VE‐cadherin (Abcam). The sections were mounted in neutral gum and visualized under a Zeiss LSM 880 confocal microscope (Carl Zeiss). The fluorescence intensity of the protein was analyzed using zen2.1/zen2 software (Carl Zeiss Microscopy GmbH). To calculate the mean vessel intensity, the sums of the pixel intensities per vessel were divided by the total vessel area (mm^2^). The mean vessel fluorescence intensity (MFI) from five images per specimen was computed and compared between the groups.

### 2.12 Popliteal LNM model

Female nude mice (4 weeks old) were purchased from the Experimental Animal Centre, Southern Medical University (Guangzhou, China). The studies were approved by the Institutional Animal Research Ethics Committee of Southern Medical University. Before tumor inoculation, we pretreated nude mice by injecting 10–15 μL recombinant human periostin (50 ng·mL^−1^) or serum‐free media (SFM) subcutaneously into the footpads for 2 weeks daily. After 2 weeks of induction, SiHa‐mCherry cells were injected into the footpads of the mice. Tumor size (mm^3^) was measured twice a week and calculated by the formula: Volume = (width)^2^ × length/2. The mice were euthanized when the primary tumors were approximately 150 mm^3^. The number of metastases was tracked in the living mice by optical imaging of mCherry activity using the *In Vivo* FX PRO system (Bruker, Billerica, MA, USA). The popliteal LNs were paraffin embedded and analyzed for mCherry expression by IHC with anti‐mCherry antibody (Abcam). Positive LNs were identified by detecting mCherry staining under a Nikon upright microscope. The ratio of metastasis positive to total dissected popliteal LN was calculated.

### 2.13 *In vitro* dextran permeability assay

The HDLECs (1 × 10^5^ cells·mL^−1^) were seeded onto gelatin‐coated Transwell^®^ filters (8 μm pore size; BD Biosciences, San Jose, CA, USA) and allowed to grow to confluence for two days in complete ECM. The CM from various fibroblasts, medium alone (ECM or 2% FBS), and recombinant human periostin (Sino Biological Inc., Beijing, China) was added to the upper chamber of the Transwell^®^. After 24 h, 10 μg·mL^−1^ FITC‐dextran (MW 70 kDa; Sigma‐Aldrich, St. Louis, MO, USA) was added to the top well. Simultaneously, the fluorescence intensity in the bottom well was monitored by measuring 50 μL medium aliquots in a time course using a Synergy™ Neo2 Multi‐Mode Reader (BioTek Instruments, Inc., Winooski, VT, USA) at 492 nm excitation and 520 nm emission [22]. The experiments were performed in triplicate; hence, the data were representative of three independent experiments.

### 2.14 Transendothelial migration assay

The HDLECs (1 × 10^5^ cells·mL^−1^) were seeded onto gelatin‐coated Transwell^®^ filters (8 μm pore size; BD Biosciences) and allowed to grow to confluence for 2 days in complete ECM. The CM from various fibroblasts, medium alone (ECM or 2% FBS), and recombinant human periostin (Sino Biological) were added to the upper chamber of the Transwell^®^, respectively. After 24 h, serum‐starved SiHa‐mCherry cells (8 × 10^4^ per filter) were seeded onto the top well. After another 24 h, the cells on the top side of the inserts were scraped off and the Transwell^®^ filters were examined for invading SiHa‐mCherry under a fluorescent microscope (Olympus Corporation, Tokyo, Japan) [10]. The experiments were performed in triplicate; hence, the results represented three independent experiments.

### 2.15 Lymphatic vessel permeability assay

After two weeks of periostin or SFM treatment, 80 mg·kg^−1^ FITC‐dextran was injected subcutaneously into mice footpads (MW 70 kDa; Sigma) 30 min before sacrifice. Fresh popliteal LNs were harvested, placed in casting molds filled with OCT (Tissue‐Tek, Sakura Finetek Europe BV, the Netherlands), and frozen in an isopentane/dry ice bath. The LNs were sectioned into 10 mm pieces on a Leica CM 1850 microtome (Leica Microsystems, Wetzlar, Germany), stained with DAPI, and observed under a fluorescence microscope (Olympus) [23, 24].

### 2.16 Microfluidic‐based 3D tumor assay for HDLEC intravasation

We created a 3D ECM (2.5 mg·mL^−1^ collagen type I) seeded with SiHa‐mCherry cells (0.8 × 10^6^ cells·mL^−1^) in a gel channel and placed the gel‐filled chips into a 37 °C incubator. Half an hour after the cell‐ECM mixture was injected into the device, we seeded the HDLEC‐GFP (1.5 × 10^6^ cells·mL^−1^) into the media channel and it was allowed to form an endothelial monolayer for 48 h. The cells were allowed to interact for another 48 h. Live cell imaging was performed to confirm the occurrence of intravasation in real time, while image stacks were visualized using a Zeiss LSM 880 with Airyscan (Carl Zeiss). We quantified the number of tumor cells that passed through the HDLECs and entered the media channel; each cell was considered as an intravasation cell [25]. The percentage of cancer cells that intravasated across the HDLEC monolayer was calculated at the end of the experiment.

### 2.17 Transmission electron microscopy

The HDLECs treated with or without periostin were fixed in 2% glutaraldehyde for 5 min. The cells were then collected and pelleted to be processed for electron microscopy. The samples were postfixed in osmium tetroxide, dehydrated in ethanol, treated with propylene oxide, and embedded in Spurr's epoxy resin. Subsequently, 90‐nm sections were stained with uranyl acetate and lead citrate prior to examination under the H‐7500 transmission electron microscope (TEM; Hitachi, Tokyo, Japan) at 10 000× and 40 000× magnification [26]. Representative images for each sample were taken from three different fields of vision.

### 2.18 Public data analysis

Gene expression data were obtained from The Cancer Genome Atlas (TCGA) databases (https://portal.gdc.cancer.gov/). Kaplan–Meier survival analyses were carried out to compare the different expression levels of periostin in 246 cervical cancer patients, based on the median expression level (expression level = 90 060.14) of periostin in TCGA mRNA‐Seq data, and patients were classified into periostin‐positive group (expression level ≥ 90 060.14) and periostin‐negative group (expression level < 90 060.14) (123 patients with negative expression levels of periostin and 123 patients with positive expression levels of periostin).

### 2.19 Statistical analysis

Statistical analysis was performed using spss v.20.0 software (SPSS Inc., Chicago, IL, USA). The data are expressed as mean ± standard deviation (SD). One‐way ANOVA was used for the comparisons among groups, and chi‐squared test (χ^2^‐test) was applied to the categorical variables. Correlation analysis was performed using Spearman's rank test. Differences were considered statistically significant at *P* < 0.05.

## 3 Results

### 3.1 CAFs derived from CSCC with LNM increased the permeability of lymphatic endothelial monolayers

**Fig. 1:**
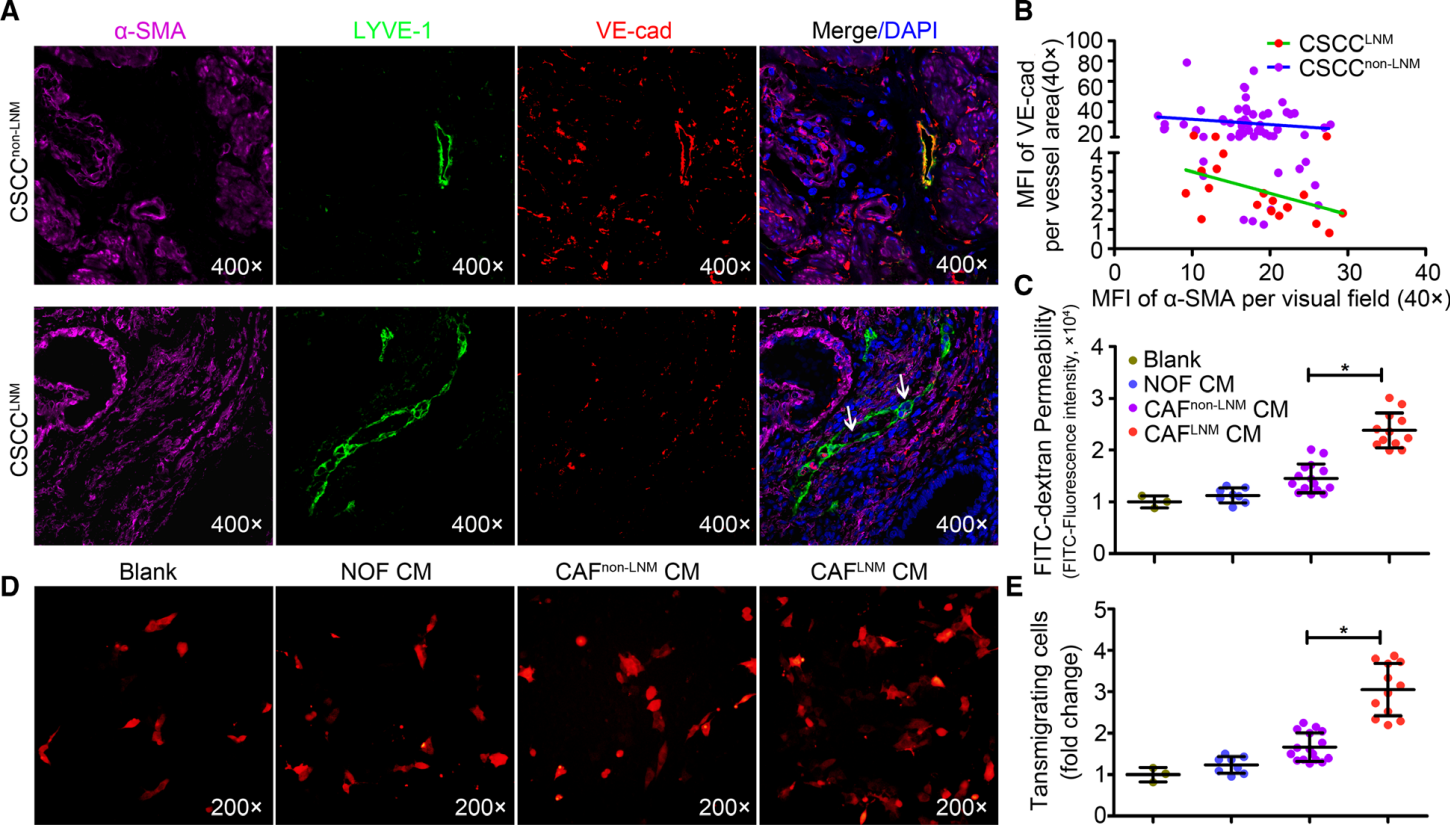
CAFs derived from CSCC with LNM increased the permeability of lymphatic endothelial monolayers. (A) Representative images of the LYVE‐1 (green), VE‐cadherin (red), and α‐SMA (purple) fluorescence staining in CSCC (20 samples with LNM; 57 samples without LNM) under 400× magnification. Blue stains indicate the cell nuclei. White arrows indicate the cancer cells invading the LVs. (B) The correlation between VE‐cadherin per LYVE‐1^+^vessel and CAFs (marker with α‐SMA). (C) Permeability was measured by the appearance of FITC‐dextran after its addition to the top well during the 1‐h time course. (D) The transmigrated SiHa‐mCherry cells on the bottom side of the filters were quantified using a fluorescent microscope. (E) Data are expressed as mean ± SD of three experiments performed in triplicate. *Significant at*P < *0.05.

VE‐cadherin, the main component of AJs, plays an important role in maintaining the integrity of intercellular barriers [27, 28]. To determine the effect of CAFs on the lymphatic endothelial barrier in the TME of CSCC, quadruple immunofluorescent staining was performed on the primary tumor samples obtained from 77 CSCC patients (consisting of 57 CSCC patients without LNM [CSCC^non‐LNM^] and 20 with LNM [CSCC^LNM^]), using α‐SMA (CAF marker), LYVE‐1 (lymphatic marker), VE‐cadherin, and DAPI (nuclear marker). The results showed that the VE‐cadherin signal was detected mainly in the lymphatic vessels (Fig. 1A). Compared to that in CSCC^non‐LNM^, the expression of VE‐cadherin was significantly reduced in CSCC^LNM^‐associated LVs. In addition, lower VE‐cadherin expression was significantly associated with lymphovascular space invasion (Fig. 1A). High density of CAFs was a common feature within the TME in both CSCC^non‐LNM^ and CSCC^LNM^ tissues, although there was no statistical difference in the number of CAFs to distinguish LNM tissue (*P* = 0.3448; Fig. 1A, Fig. S1). However, the density of CAFs was inversely associated with VE‐cadherin expression in CSCC^LNM^ tissues (*r* = −0.4512, *P* = 0.0458). In contrast, there was no correlation between the two in CSCC^non‐LNM^ tissues (*r* = −0.1655, *P = *0.2186; Fig. 1B).

To confirm the presence of heterogeneous CAFs in CSCC^non‐LNM^ and CSCC^LNM^, we isolated normal fibroblasts (NOFs) from eight normal cervical tissues, and CAFs from fifteen CSCC non‐LNM (CAF^non‐LNM^) and twelve CSCC^LNM^ (CAF^LNM^) tissues. The NOFs were positive for the expression of vimentin and negative for the expression of α‐SMA, FAP, pan‐CK, and CD31, whereas both CAFs^non‐LNM^ and CAFs^LNM^ were positive for the expression of vimentin, α‐SMA, and FAP, and negative for the expression of pan‐CK and CD31 (Fig. S2), with no difference between the two CAF types in the expression levels of all markers. Next, we performed permeability assays using a modified Transwell^®^ model and FITC‐dextran to determine the association between CAFs and disruption of lymphatic endothelial barrier. FITC‐dextran showed significant transendothelial permeability in the CAF^LNM^‐CM compared with the CM from the other groups (*P* < 0.001; Fig. 1C). Furthermore, transendothelial migration assay was used to mimic the cancer cell intravasation/extravasation process *in vitro*. The transendothelial migration of SiHa‐mCherry in the CAF^LNM^‐CM‐treated group showed a 3.05‐fold increase compared with the migration in the control group (*P* < 0.001; Fig. 1D,E). However, treatment with CAF^non‐LNM^‐CM also resulted in slightly increased permeability, but the difference was not significant when compared to that of the control and NOF‐CM‐treated groups (*P* > 0.05). Collectively, these results suggest that the CAFs from CSCC^non‐LNM^ and CSCC^LNM^ contain functionally distinct subtypes and that the CAF^LNM^ subset enhances the permeability of lymphatic endothelial monolayers in the TME.

### 3.2 Periostin is highly expressed in CAFs derived from CSCC with LNM

**Fig. 2:**
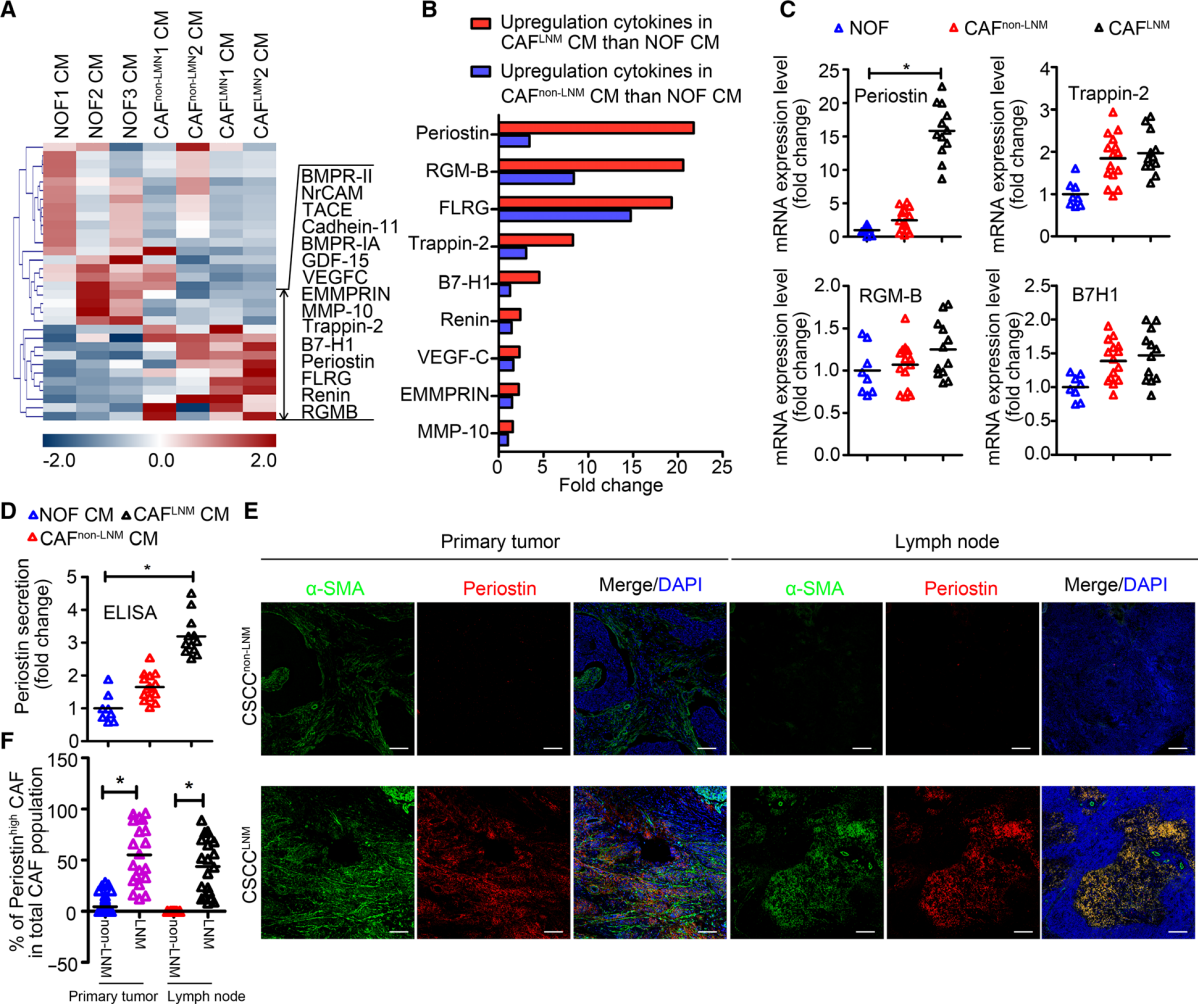
Periostin is highly expressed in CAFs derived from CSCC with LNM. (A) Expression profiles of the cytokines in the NOF‐CM, CAF^non‐LNM^‐CM, and CAF^LNM^‐CM. (B) Significantly upregulated cytokines in CAF^non‐LNM^‐CM and CAF^LNM^‐CM compared with NOF‐CM. (C) Expression of four cytokines that were significantly different between CAF^non‐LNM^and CAF^LNM^in all primary fibroblasts. (D) Confirmation of periostin expression in all primary fibroblasts. (E) Representative images of CSCC tissues and matched LNs stained for α‐SMA (green) and periostin (red). Blue stains indicate the cell nuclei. (F) Percentage of periostin^+^CAFs in total α‐SMA^+^CAFs of CSCC and paired LN samples with/without LNM (20 samples with LNM; 57 samples without LNM). Scale bar, 100 μm. *Significant at*P < *0.05.

Recent studies have reported that the CAF secretome directly regulates tumor progression and that different CAF subsets have distinct cytokine expression patterns [29]. To identify the factors involved in CAF^LNM^‐mediated impairment of the lymphatic endothelial barrier, a cytokine antibody array was performed. We searched for differentially expressed cytokines and found that periostin, RGM‐B, Trappin‐2, and B7H1 were significantly upregulated in CAF^LNM^ (Fig. 2A,B). Notably, periostin was validated to be the most significantly upregulated cytokine by qPCR (NOFs *vs*. CAFs^non‐LNM^ *vs*. CAFs^LNM^ = 1 : 1.65 : 3.12) and ELISA (NOFs *vs*. CAFs^non‐LNM^ *vs*. CAFs^LNM^ = 1 : 2.46 : 15.84) (*P* < 0.05; Fig. 2C,D, Fig. S3).

To examine the expression pattern of periostin in CSCC tissues, 57 CSCC^non‐LNM^ and 20 CSCC^LNM^ tissues and their paired LNs were subjected to triple immunofluorescent staining of α‐SMA, periostin, and DAPI (Fig. 2E). The results showed colocalization of periostin and α‐SMA in the stromal regions of the CSCC tissues, confirming that periostin was preferentially expressed by CAFs (Fig. 2F). We designated the α‐SMA^+^cells with high periostin expression as periostin^+^CAF and the cells with low or no periostin expression as periostin^‐^CAF. The percentage of periostin^+^CAFs was significantly higher in CSCC^LNM^ tissues (12–96%, *n* = 20) than in CSCC^non‐LNM^ tissues (0–28%, *n* = 57, *P* < 0.05; Fig. 2F). Similarly, the proportion of periostin^+^CAFs was significantly higher in the LNs from CSCC^LNM^ (8–89%, *n* = 20, *P* < 0.05; Fig. 2F) than in those from CSCC^non‐LNM^ (almost undetectable, *n* = 57), indicating that the immunostained cells were subsets of LNM‐associated CAFs. Collectively, our results show that the abundance of periostin^+^CAFs in the TME is crucial for LNM of CSCC. Periostin was selected for further analysis because of its recognized role in mediating the crosstalk between CAFs and TME [30, 31].

### 3.3 Periostin downregulates VE‐cadherin and impairs the barrier function of lymphatic endothelial monolayers *in vitro*

**Fig. 3:**
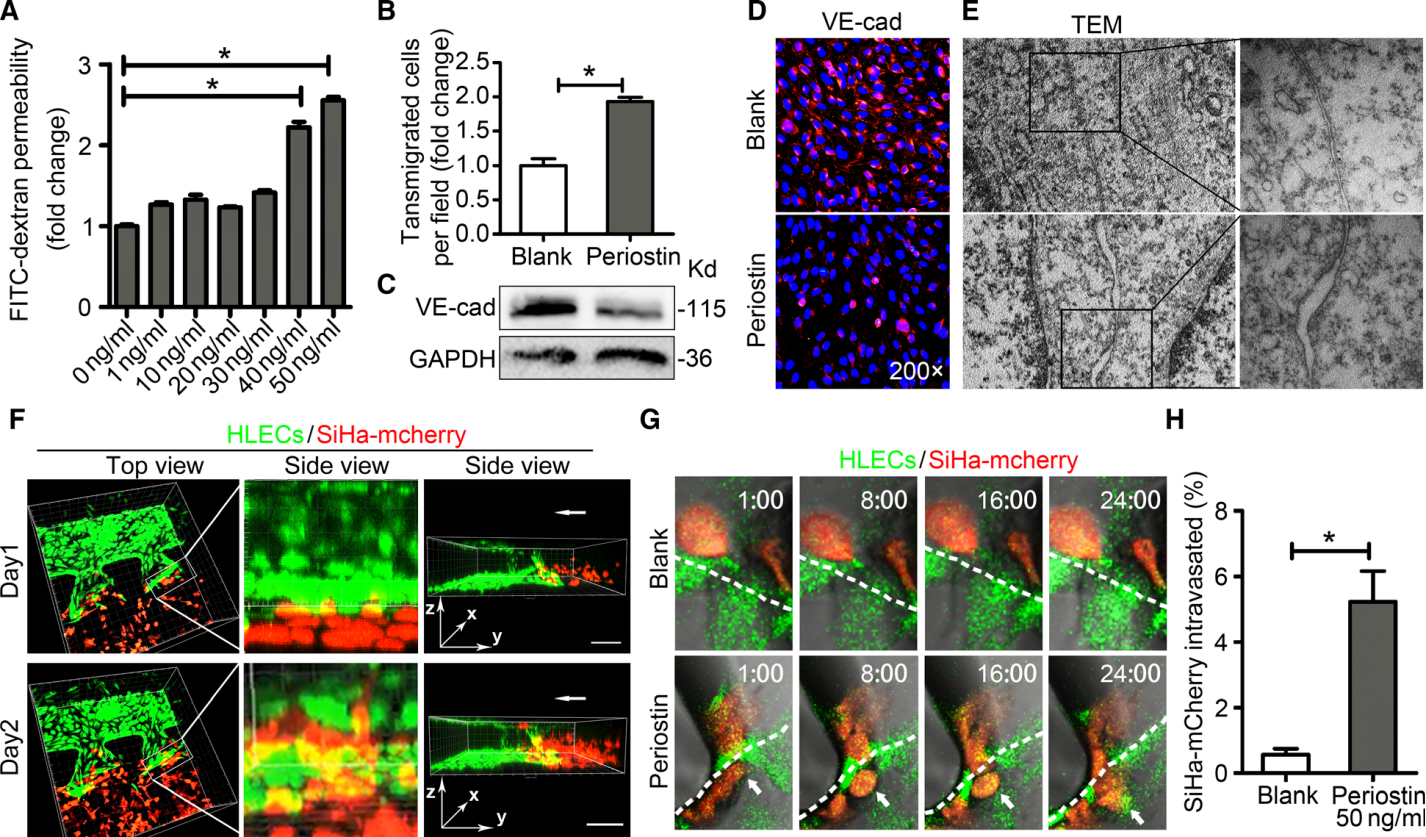
Periostin downregulates VE‐cadherin and impairs the barrier function of lymphatic endothelial monolayers*in vitro*. (A) Permeability of HDLECs treated with recombinant periostin at different concentrations (0, 1, 10, 20, 30, 40, and 50 ng·mL^−1^) for 24 h. (B) Confluent HDLEC monolayers in the untreated and periostin‐treated (50 ng·mL^−1^) groups after 24 h. SiHa‐mCherry cells were seeded onto the monolayers for another 24 h, and the transmigrated SiHa‐mCherry cells were quantified. (C) Western blot results of VE‐cadherin in periostin‐treated HDLECs. (D) HDLEC monolayers incubated with or without periostin (50 ng·mL^−1^) for 24 h were analyzed by immunofluorescence (IF) staining of VE‐cadherin (red). Blue stains indicate the cell nuclei. (E) TEM images showing the lymphatic endothelial cell–cell junctional integrity in the untreated and periostin‐treated (50 ng·mL^−1^) groups (left panels are under 10 000× magnification; right panels are under 40 000× magnification). (F) Three‐dimensional rendering of the hydrogel region showing the periostin‐treated (50 ng·mL^−1^) lymphatic endothelial monolayer (green) next to the SiHa‐mCherry cells (red) at days 1 and 2. Scale bar at 200 μm. (G) Upper panels show that the SiHa‐mCherry cells (red) did not pass through lymphatic endothelial monolayer (green). Lower panels show that the SiHa‐mCherry cell (red) invaded the periostin‐stimulated lymphatic endothelial monolayer (green). (H) Percentage of SiHa‐mCherry cells that intravasated across the HDLECs monolayer. Data are expressed as mean ± SD of three experiments performed in triplicate. *Significant at *P < *0.05.

Exogenous recombinant human periostin was used to mimic the functional role of periostin in HDLECs that was detected using *in vitro* dextran permeability assay. Treatment with recombinant human periostin significantly increased the movement of FITC‐dextran probes to the bottom wells (Fig. 3A). The highest rate of permeability was observed in the HDLEC monolayer treated with 50 ng·mL^−1^ periostin (*P* < 0.05). Likewise, the number of SiHa‐mCherry cells that migrated across the HDLEC monolayer was significantly greater in the periostin‐treated group (1.93 ± 0.10) compared with the untreated group (1 ± 0.17) (*P* < 0.05; Fig. 3B). Western blotting and immunofluorescence analyses of the HDLEC monolayers revealed that treatment with periostin resulted in markedly reduced VE‐cadherin levels (Fig. 3C,D). However, the expression levels of other junction proteins, such as ZO‐1, ZO‐2, and occludin, did not change significantly (Fig. S4). Transmission electron microscopy revealed intact tight junctions between the HDLECs in the untreated group. Conversely, the cell–cell junctions were disrupted with increased spaces between adjacent cell membranes in the periostin‐treated HDLEC monolayers (Fig. 3E).

To directly observe transendothelial migration of cancer cells, we performed a microfluidic‐based 3D tumor assay for HDLEC intravasation that enabled the real‐time visualization and quantification of the interactions between cancer cells and HDLEC monolayer that were stimulated with 50 ng·mL^−1^ periostin for 2 days. The invasion of SiHa‐mCherry cells across the lymphatic endothelial barrier was observed 24 h after barrier formation in the periostin‐treated group, while no cell invasion was observed in the untreated group (Fig. 3F,G, Video S1). After 48 h, intravasation occurred for a small fraction of the cancer cells in contact with the HDLEC monolayer in the untreated group (0.57 ± 0.18%). However, a significantly higher percentage of SiHa‐mCherry cells intravasated following periostin treatment (5.22 ± 0.93%) (*P* < 0.05; Fig. 3H). These findings suggest that impaired lymphatic endothelial barrier function facilitates cancer cell intravasation and that periostin‐induced lymphatic endothelial barrier disruption promotes transendothelial migration of cancer cells.

### 3.4 Periostin induces lymphatic permeability and promotes metastasis *in vivo*

**Fig. 4:**
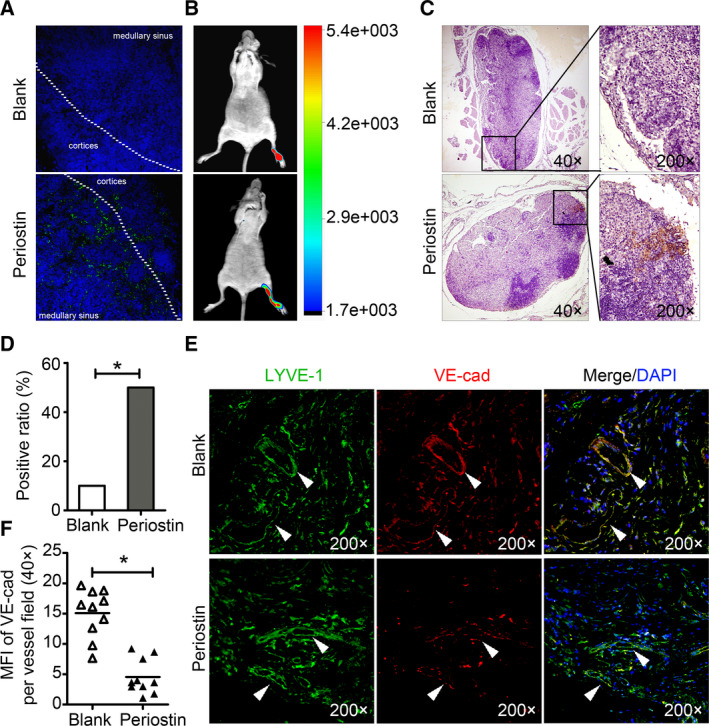
Periostin induces lymphatic permeability and promotes metastasis*in vivo*. (A) Representative images of the*in vivo*lymphatic vessel permeability assay in mouse footpad injected subcutaneously with FITC‐dextran (green) (*n* = 3). Blue stains indicate the cell nuclei. (B)*In vivo*fluorescence images of lymphatic metastasis. (C) Staining of mCherry in popliteal lymph nodes from mice. Metastasis positive lymph nodes (LNs) were identified by staining for cancer cell‐expressed mCherry. (D) Ratio of the metastasis positive LNs to the total dissected popliteal LNs in mice treated with the indicated CM. *Significant at *P < *0.05. (E) Representative images of the paraffin sections from the tumor of experimental mice stained with both anti‐LYVE‐1 (green) and anti‐VE‐cadherin (red) antibodies under 400× magnification. White triangles indicate the LVs. (F) Mean vessel fluorescence intensity (MFI) of VE‐cadherin per vessel field in experimental mice. *Significant at *P < *0.05.

To further demonstrate the *in vivo* effect of periostin on lymphatic endothelial barrier, 10–15 μL periostin (50 ng·mL^−1^) or PBS were injected subcutaneously into mice footpads daily for 2 weeks, following which the diffusion of large molecules (FITC‐dextran, 70 kDa) in the LNs was examined. The results showed that FITC‐dextran was dispersed in the cortices and medullae of the LNs in periostin‐treated mice compared with the untreated mice (Fig. 4A). In addition, periostin‐pretreated SiHa‐mCherry cells were subcutaneously injected into the mice footpads. Consistent with the effect of enhancing the permeability of lymphatic endothelial barriers, periostin significantly promoted lymphatic metastasis *in vivo*, at a ratio of 50% and 10% LNM in the periostin‐treated and untreated groups, respectively (Fig. 4B–D, Table S2), accompanied by reduced VE‐cadherin levels in the LVs (Fig. 4E,F). Collectively, these results suggest that high periostin levels in the TME increase the metastatic potential by weakening the lymphatic endothelial barrier in the host.

### 3.5 Periostin‐induced impairment of lymphatic endothelial barrier function is driven by the integrin‐FAK/Src pathway

**Fig. 5:**
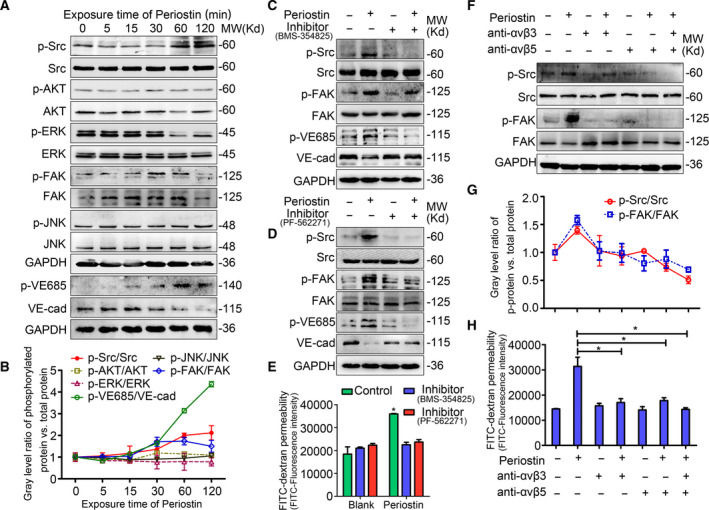
Periostin‐induced impairment of lymphatic endothelial barrier function is driven by the integrin‐FAK/Src pathway. (A) Western blot analysis of the signaling pathways activated in periostin‐treated HDLECs. (B) Line chart showing the changes (indicated by the gray level) in the ratio of phosphorylated protein to total protein as described in A. (C) Western blot analysis of the activation of Src and FAK in HDLECs untreated or treated with BMS‐354825 monohydrate (Src inhibitor) for 1 h before incubation with or without periostin for 1 h. (D) Western blot analysis of the activation of Src and FAK in HDLECs untreated or treated with PF‐562271 (FAK inhibitor) for 1 h before incubation with or without periostin for 1 h. (E) Permeability of HDLECs was analyzed following the treatments described in C and D. (F–H) HDLECs were pretreated with/without blocking antibodies against integrins αvβ3 (R&D, MAB3050, 2 μg·mL^−1^) or αvβ5 (R&D, MAB2528, 10 μg·mL^−1^), and then treated with periostin for 1 h. (F) Western blot analysis of phospho‐Src and phospho‐FAK in HDLECs. (G) Line chart showing the changes in the ratios of phospho‐Src to total Src, and phospho‐FAK to total FAK. (H). Permeability of HDLECs following the indicated treatments.

Periostin regulates certain biological functions by activating downstream signaling cascades in recipient cells [32]. We performed western blotting to analyze the periostin‐related signaling pathways involved in lymphatic endothelial function included the Src, AKT, ERK1/2, FAK, and JNK pathways. Incubation of HDLECs with periostin resulted in the activation of integrin signaling, as evidenced by an increase in phosphorylated‐FAK (p‐FAK, Tyr397) and Src (p‐Src, Tyr416) (Fig. 5A,B, Fig. S5A). Other signaling pathway indicators, including AKT, ERK, and JNK, were not activated at up to 120 min after periostin treatment. The inhibition of FAK signaling using PF‐562271 clearly inhibited the periostin‐induced activation of FAK and Src. In contrast, the inhibition of Src signaling using BMS‐354825 monohydrate did not suppress the periostin‐induced activation of FAK (Fig. 5C,D, Fig. S5B,C). These results suggest that periostin‐induced FAK phosphorylation is required for Src kinase activity. VE‐cadherin has been reported to be a direct substrate of Src [33]. Consistent with this, we showed that the ratio of tyrosine‐phosphorylated VE‐cadherin to VE‐cadherin gradually increased following the activation of FAK/Src. The inhibition of FAK/Src alleviated the tyrosine‐phosphorylation of VE‐cadherin and further increased VE‐cadherin levels (Fig. 5C,D, Fig. S8), suggesting that periostin‐induced activation of FAK/Src promoted VE‐cadherin degradation directly. Assays for transendothelial migration and *in vitro* permeability demonstrated that the administration of BMS‐354825 monohydrate and PF‐562271 prevented periostin‐induced hyperpermeability of the lymphatic endothelial barrier (Fig. 5E, Fig. S6). Similarly, activation of FAK/Src signaling was observed in HDLECs cells 60 min after treatment with periostin^+^CAF‐CM. Furthermore, the functional association of the FAK/Src signaling pathway with the periostin^+^CAF‐CM‐induced lymphatic endothelial permeability was also blocked by BMS‐354825 monohydrate and PF‐562271 (Fig. 5E, Fig. S6).

Because periostin is a ligand for several integrins including αvβ3 and αvβ5 [34], we sought to determine the relationship between periostin‐dependent FAK/Src activation and integrin binding. After confirming the presence of αvβ3 and αvβ5 in the HDLECs [35], we found that in response to periostin and periostin^+^CAF‐CM, FAK/Src phosphorylation was suppressed by the blocking antibodies directed against both αvβ3 and αvβ5 (Fig. 5F,G, Fig. S5D). Correspondingly, the blockade of αvβ3 and αvβ5 in HDLECs significantly abrogated the cell hyperpermeability stimulated by periostin^+^CAF‐CM (Fig. 5H, Fig. S7), indicating that the αvβ3/αvβ5‐FAK/Src axis is required for the regulation of the periostin^+^CAF‐CM‐induced lymphatic monolayer permeability. Collectively, these results demonstrate that the interaction of periostin^+^CAF‐secreted periostin with endothelial αvβ3 and αvβ5 triggers FAK/Src signaling, consequently promoting lymphatic endothelial permeability and cancer cell intravasation.

### 3.6 High periostin expression correlates with low VE‐cadherin expression and poor survival in CSCC patients

**Fig. 6:**
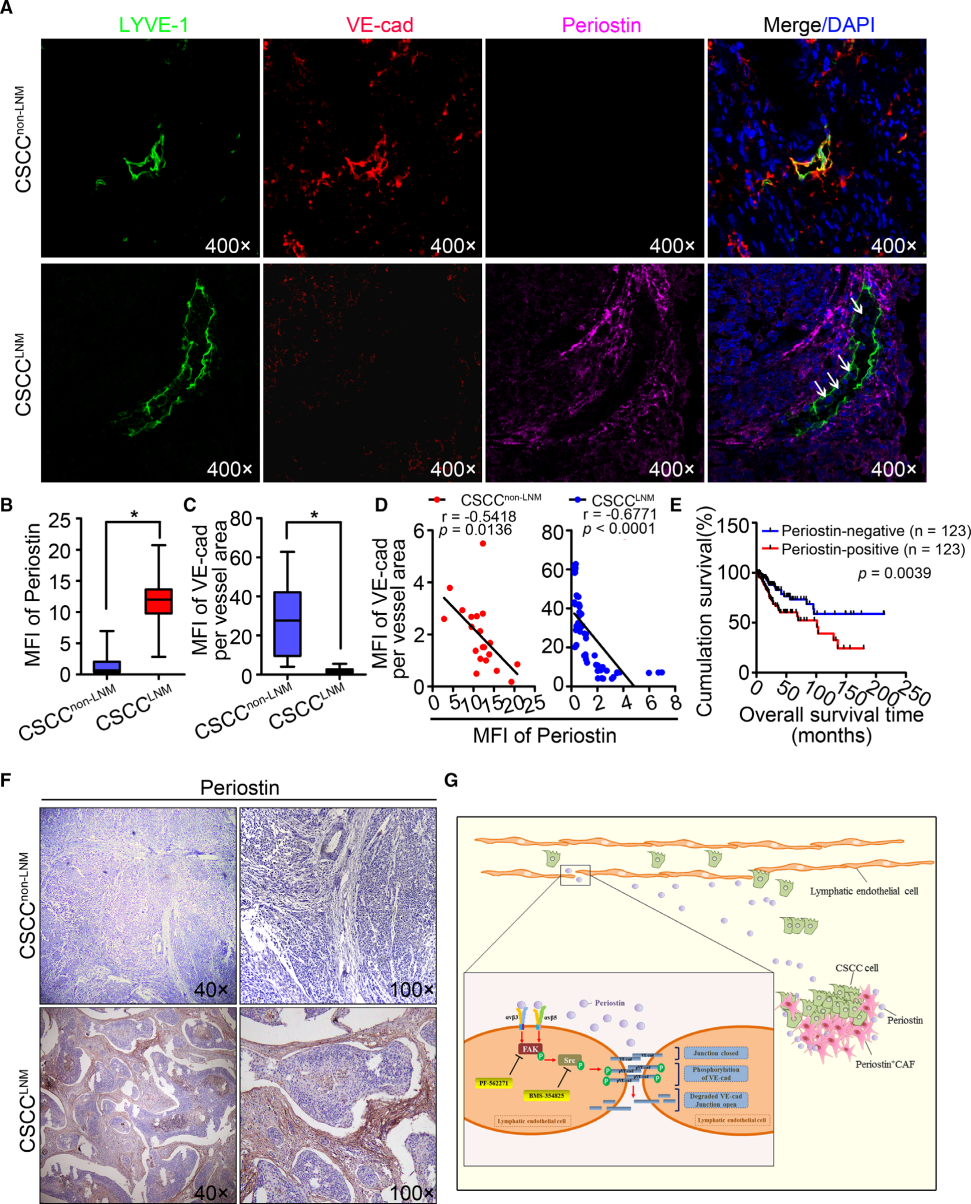
High periostin expression correlates with low VE‐cadherin expression and poor survival in CSCC patients. (A) Representative images of CSCC tissues stained for LYVE‐1 (green), VE‐cadherin (red), or periostin (purple). Blue stain indicates the cell nuclei. White arrows indicate cancer cells that have invaded into the LVs. (B) Periostin fluorescence intensity per area of CSCC tissues. Results are presented as mean ± standard deviation (SD). *P < 0.05. (C) VE‐cadherin fluorescence intensity per LVs of CSCC tissues. Results are presented as mean ± SD. *P < 0.05. (D) The correlation between periostin and VE‐cadherin was analyzed in both CSCC^LNM^(*n* = 20) and CSCC^non‐LNM^(*n* = 57). (E) Kaplan–Meier survival curves for cervical cancer patients with periostin‐high versus periostin‐low tumors in TCGA online database (*n* = 246). (F) Representative images of periostin staining in CSCC specimens. (G) Schematic model: periostin secreted by periostin^+^CAFs results in dysfunction of lymphatic endothelial barriers by activating integrin‐FAK/Src signaling pathway in lymphatic endothelial cell. Blocking periostin or its downstream effectors (using inhibitors) FAK (PF‐562271)/Src (BMS‐354825 monohydrate) may inhibit periostin^+^CAF‐induced LNM.

To clarify the relationship between VE‐cadherin expression, lymphatic vessels, and stromal periostin in CSCC, primary tumor samples derived from CSCC were examined using quadruple immunofluorescent staining for periostin, LYVE‐1, VE‐cadherin, and DAPI (Fig. 6A). Compared to that in CSCC^non‐LNM^ group (*n* = 57; Fig. 6B), stromal periostin expression was significantly higher in the CSCC^LNM^ group (*n* = 20; Fig. 6B). Moreover, intense periostin staining was observed around VE‐cadherin^low^ LVs, whereas periostin expression was lower around VE‐cadherin^high^ LVs (Fig. 6C, Fig. S8), indicating that decreased VE‐cadherin expression in the LVs was observed in the periostin^+^CAF‐enriched regions of CSCC. Correspondingly, stromal periostin expression was inversely correlated with VE‐cadherin expression in the LVs both in CSCC^LNM^ (*r* = −0.5418, *P* = 0.0136, Fig. 6D) and in CSCC^non‐LNM^ (*r* = −0.6771, *P* < 0.0001; Fig. 6D).

Periostin expression in CSCC specimens was further analyzed using immunohistochemistry to determine the clinical relevance of our findings (Fig. 6F). Statistical analysis revealed no significant correlation between periostin expression and pathology grade (*P* = 0.080), parametrial involvement (*P* = 0.190), and age (*P* = 0.650). In contrast, the expression level of stromal periostin was significantly correlated with lymphatic metastasis (*P* = 0.001), FIGO stage (*P* = 0.046), and vascular invasion (*P* = 0.006) in CSCC patients (Table 1). Consistent with the immunostaining analysis, high expression of periostin genes was significantly associated with shorter overall survival in the TCGA dataset (*P* = 0.0039; Fig. 6E), indicating that the upregulation of periostin expression is correlated with poor outcome and shorter survival in CSCC patients.

**Table 1:** Association between Periostin expression and clinicopathologic features in 77 patients with CSCC.

Group	N	Periostin		High expression percentage (%)	χ^2^ (*P*)
		Low	High		
FIGO stage					
Early CSCC (I–IIa)	62	59	3	4.84	3.98 (0.046)
Lately CSCC (IIb–IV)	15	3	12	80.00	
Lymphatic metastasis					
No	57	52	5	8.77	10.20 (0.001)
Yes	20	11	9	45.00	
Pathology grade					
1	39	21	17	43.59	5.019 (0.080)
2	32	22	10	31.25	
3	6	4	2	33.33	
Vascular invasion					
No	69	45	24	34.78	8.284 (0.006)
Yes	8	1	7	87.50	
Parametrial involvement					
No	73	51	22	30.14	1.74 (0.190)
Yes	4	1	3	75.00	
Age					
< 45	25	16	9	36.00	0.21 (0.650)
≥ 45	52	36	16	30.77	

In summary, periostin^+^CAFs impair lymphatic endothelial barrier by activating the integrin‐FAK/Src signaling pathway in lymphatic endothelial cells. The mechanisms presented in this study are summarized in a schematic diagram (Fig. 6G).

## 4 Discussion

Cancer‐associated fibroblasts, which constitute the most abundant and heterogeneous stromal cells in the TME, are critically involved in cancer progression [3]. They closely interact with other cells within the TME and actively promote cancer progression by secreting cytokines that activate the signaling pathways involved in tumor cell proliferation and metastasis [4, 36]. Tumors with high CAF density are common in CSCC [37, 38]; however, only a small proportion of tumors have lymphatic metastasis. Consistent with previous reports, we confirmed that CSCC^LNM^ and CSCC^non‐LNM^ tissues contain functionally distinct CAF subtypes. Therefore, therapeutic strategies that target the whole CAF population are likely to be ineffective due to fibroblast heterogeneity and may even contribute to cancer progression [3]. Our study identified a novel metastatic‐promoting CAF subset and investigated its clinical significance in CSCC. We discovered that periostin^+^CAF promoted LNM by impairing the lymphatic endothelial barrier function. Interestingly, periostin^+^CAF was rarely found in CSCC^non‐LNM^ tissues, which may explain their low metastatic potential. Moreover, periostin^+^CAF abundance in the TME appears essential for tumor cell dissemination, potentially contributing to the biological differences between CSCC^LNM^ and CSCC^non‐LNM^ tissues. Thus, we provide mechanistic and clinical insights into the role of periostin^+^CAF in the LNM of CSCC.

In the present study, we found significant accumulation of periostin^+^CAFs in both primary tumors and metastatic LNs from CSCC. Periostin is a secreted matricellular protein that plays an important role in tissue remodeling and collagen fibrillogenesis by interacting with ECM proteins, such as fibronectin and collagen V, and cell surface receptors [39]. It is typically absent in normal adult tissues, but is highly expressed in injured tissues and tumor stroma [39]. In addition, periostin contributes to the creation of a pro‐metastatic niche to support metastasis [31]. In the early phase of pro‐metastatic niche formation, tissue responses are accompanied by dysfunction of the endothelial cell barrier [40]. Our data suggest that periostin potentially mediates the crosstalk between the CAFs and lymphatic endothelial cells (LECs) in TME that induces the disruption of lymphatic endothelial barriers. The disruption of endothelial barrier integrity, frequently resulting in increased permeability to cells and solutes, is a critical step for cancer cells to enter the LV and migrate to LNs [41]. The endothelial barrier integrity is partly regulated by the dynamic opening and closure of intercellular AJs. AJs are largely composed of VE‐cadherin, an endothelium‐specific member of the cadherin family of adhesion proteins that binds to several protein partners, including p120, β‐catenin, and plakoglobin, via the cytoplasmic domain [42]. Alteration in the cellular localization of VE‐cadherin and its dissociation from the actin cytoskeleton is associated with increased LV permeability [33, 43]. In addition, Zhou *et al*. [22] provided evidence that enhanced LV permeability facilitates cancer cell dissemination and growth at distant sites through multiple methods, such as the leakage of large proteins, resulting in a higher concentration of tumor cells. In this study, we demonstrated that periostin significantly decreased VE‐cadherin expression, disrupted the intercellular junctions of the lymphatic endothelial barrier, and opened the intercellular spaces between the endothelial cells, consequently facilitating transendothelial migration of cancer cells, and promoting lymphatic metastasis. Moreover, the percentage of periostin^+^CAF in the total CAF population was negatively correlated with VE‐cadherin expression in the LVs. Periostin has been reported to promote tumor cell proliferation, invasion, and migration [38, 44, 45]; however, we report its novel function in mediating lymphatic barrier breakdown through VE‐cadherin disruption.

Periostin contains an FAS1 domain that allows it to bind to integrins. Interaction with these cellular receptors leads to diverse downstream signaling effects in a context‐dependent manner [32]. For instances, periostin increases proliferation of cancer cells via activating the ERK pathway, but regulates endothelial cell function via αvβ3 signaling [39], which implies varying effects of periostin in different cells. In this study, periostin‐dependent hyperpermeability in the LECs was suppressed through dual‐blocking of αvβ3 and αvβ5, indicating that the interaction of αvβ3/αvβ5 and deposited periostin is critical for promoting lymphatic endothelial barrier dysfunction. Recently, the FAK/Src signaling complex has been recognized as a distinct functional unit that regulates integrin‐dependent cell functions [46]. Integrin‐stimulated FAK phosphorylation at Tyr397 creates a high‐affinity binding site for the Src‐homology 2 (SH2) domain of Src family kinases (SFKs), and recruits activated Src kinases to the adhesion complexes. The binding of Src to FAK leads to conformational activation of SFKs and contributes to integrin‐related signal transduction [47]. Consistently, we observed that in LECs cultured on periostin, FAK phosphorylation at Tyr397 was dependent on both αvβ3 and αvβ5 and that FAK activation was required for Src kinase activity, suggesting that the FAK/Src pathway may act as a node for periostin‐induced integrin signaling in LECs.

Src, a member of the Src kinase family, is one of the major signaling proteins associated with the loss of endothelial barrier function [48]. Wallez *et al*. [33] reported that VE‐cadherin is a direct substrate for Src kinase and that Tyr685 represents a unique phosphorylation site in the VE‐cadherin cytoplasmic domain. This research also indicated a direct involvement of Src activity in VE‐cadherin hyperphosphorylation, which correlated with the internalization and degradation of VE‐cadherin. In the present study, we revealed that the inhibition of FAK/Src kinases blocked the VE‐cadherin‐Tyr685 phosphorylation and restored the junctional VE‐cadherin expression in periostin‐treated LECs, and further nullified the hyperpermeability of LECs induced by periostin or periostin^+^CAF. Taken together, these data suggest that periostin‐induced FAK/Src activity directly regulates VE‐cadherin activation and dissociation, while the inhibition of FAK/Src mitigates these effects. Therefore, the constant accumulation of periostin^+^CAF in the TME induces FAK/Src phosphorylation by binding to αvβ3 and αvβ5 in the recipient cells. Phosphorylated‐FAK/Src further downregulates VE‐cadherin expression in LECs and increases the permeability of LVs in CSCC^LNM^.

The prognostic values of CAFs, previously identified by conventional markers such as α‐SMA or FAP, are often different or even contradictory in some studies [49]; hence, stromal expression of α‐SMA and FAP could not accurately predict LNM [49]. In this study, however, high stromal periostin expression was found to be closely associated with LNM, vascular involvement, and low overall survival in CSCC patients. Thus, accumulation of periostin^+^CAFs serves as a promising potential biomarker for LNM and a prognostic factor for poor clinical outcomes in CSCC patients. Since the discovery of CAFs in solid tumors, several strategies focused on targeting of CAFs have been presented in experimental studies [2, 14]. However, the various approaches targeting the stroma have provided contradictory results that may promote worse outcomes instead [3]. Our findings confirming heterogeneity of CAFs address these conflicting reports regarding contrasting CAF functions. For instance, attempts to deplete CAFs based on their α‐SMA expression resulted in decreased Teff/Treg ratio and significant elevation in CTLA4 expression [3, 50, 51]. Moreover, genetic disruption or prolonged pharmacological inhibition of the hedgehog signaling pathway, which is necessary for CAF activation, causes undifferentiated pancreatic ductal adenocarcinoma tumors and decreased survival in mice [50, 51]. Accordingly, therapeutic development must consider the possibility that certain CAF subtypes may be protumorigenic, whereas others may be antitumorigenic. Identifying specific markers will provide an opportunity to target metastasis‐promoting CAFs *in vivo*. Based on our results, previous CAF‐targeting strategies for cancer treatment may have preferentially eliminated periostin^+^CAFs while leaving other CAF populations intact. Further studies are needed to confirm whether eradicating periostin^+^CAFs or inhibiting the FAK/Src signaling pathway represent more efficient therapeutic modalities to eliminate LNM in CSCC.

## 5 Conclusion

In conclusion, our findings strongly corroborate the importance of periostin^+^CAF in CSCC progression and highlight the therapeutic potential of inhibiting the FAK/Src signaling pathway for effective prevention of periostin^+^CAF‐mediated hyperpermeability of the lymphatic endothelial barriers. Furthermore, the identification of periostin^+^CAF and its regulatory mechanisms offers a novel target for the development of anti‐metastatic therapies in the future.

## Conflict of interest

The authors declare no conflict of interest.

## Author contributions

W‐FW, WW, ZH, and LL designed the study. W‐FW, X‐JC, L‐JL, LY, X‐GW, C‐FZ, and Z‐CW performed the experiments. L‐SF performed computational analyses. X‐JC and L‐JL performed the Popliteal LNM model. W‐FW analyzed the human cytokine antibody array data. W‐FW, WW, ZH, and LL wrote the manuscript. WW supervised the study. ZH and LL gave insightful discussion and constructive comments on the manuscript. All authors approved the manuscript.

## Ethical approval and consent to participate

This research was approved by the Ethics Committee of Guangzhou Medical University, and informed consent was obtained from all patients before enrolling in the research program. The *in vivo* assay using nude mice was approved by the Institutional Animal Care and Use Committee of Guangzhou Medical University.

## Supporting information


**Fig. S1.** Quantification of a‐SMA immunofluorescence staining in CSCC^non‐LNM^ and CSCC^LNM^ samples (*p* = 0.3448).
**Fig. S2.** Identification of NOFs and CAFs.
**Fig. S3.** The confirmation of Trappin‐2, RGMB, B7H1 expression in all primary fibroblasts by ELISA analysis.
**Fig. S4.** Western blot analysis of ZO‐1, ZO‐2 and Occludin in HDLECs with periostin treatment.
**Fig. S5.** Periostin^+^CAFs hyperactivate integrin‐FAK/Src axis in lymphatic endothelial cells.
**Fig. S6.** Periostin‐induced hyperpermeability is driven by FAK/Src pathway.
**Fig. S7.** Periostin affects HDLECs permeability by binding to αvβ3 and αvβ5.
**Fig. S8.** The decreased expression of VE‐cadherin induced by periostin were abrogated in FAK/Src inhibitor treated monolayers.
**Fig. S9.** Representative images of CSCC^LNM^ tissues stained for LYVE‐1 (green) and VE‐cadherin (red) and periostin (purple).

**Table S1.** Primers for real‐time RT‐PCR.
**Table S2.** Effect of Periostin on popliteal lymph nodes (LNs) metastasis in vivo.

**Video S1.** Time‐lapse view of cancer cell extravasation.

## Data Availability

The datasets used and/or analyzed during the current study are available from the corresponding author on reasonable request.
