# Supplementary material for: Periostin+cancer‐associated fibroblasts promote lymph node metastasis by impairing the lymphatic endothelial barriers in cervical squamous cell carcinoma
Source: Mol Oncol. 2020 Nov 12;15(1):210–27. doi: 10.1002/1878-0261.12837 (PMC7782076; doi:10.1002/1878-0261.12837)

**Supplementary Figure 1. Quantification of a-SMA** **immunofluorescence staining in CSCC^non-LNM^ and CSCC^LNM^ samples (*p*=0.3448).**


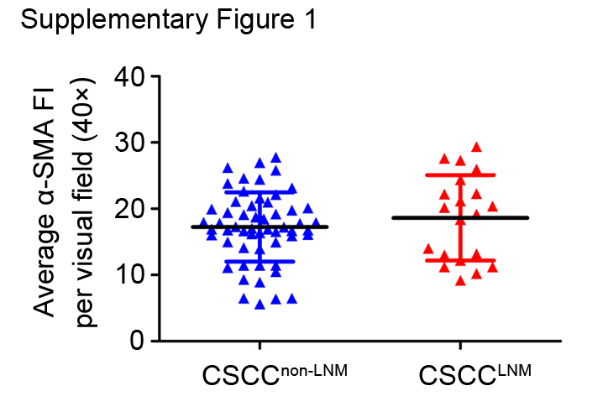


**Supplementary Figure 2. Identification of NOFs and CAFs.**

**A.** Western blot for CD-31, pan-cytokeratin, α-SMA, FAP and Vimentin in HUVECs, SiHa and primary NOFs from normal cervical and CAFs from CSCC^non-LNM^ and CSCC^LNM^. **B.** Protein level of α-SMA, FAP and Vimentin in other CAFs from CSCC^non-LNM^ and CSCC^LNM^ (Denoted as gray level ratio of target protein to GAPDH). **C.** immunofluorescence for CD-31, pan-cytokeratin, α-SMA, FAP and vimentin in NOFs and CAFs. Images are shown at×400 magnification.


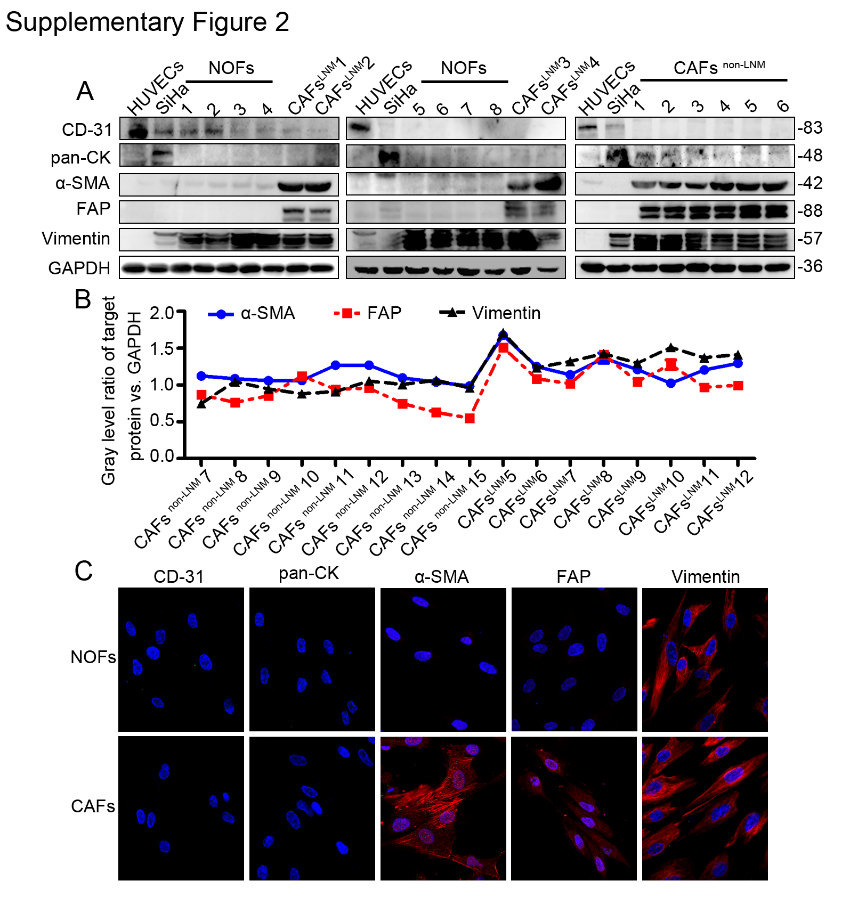


**Supplementary Figure 3. The confirmation of Trappin-2, RGMB, B7H1 expression in all primary fibroblasts by ELISA analysis.**


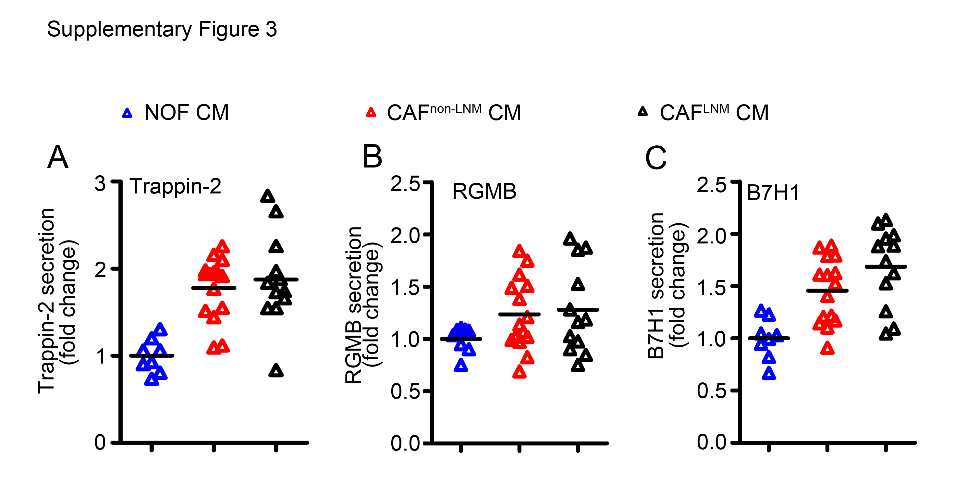


**Supplementary Figure 4. Western blot analysis of ZO-1, ZO-2 and Occludin in HDLECs with periostin treatment.**


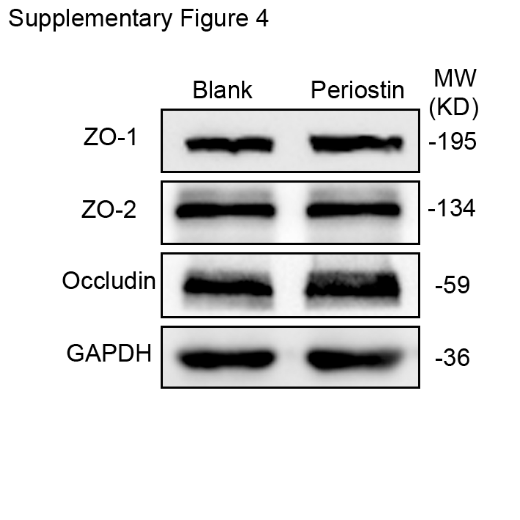


**Supplementary Figure 5. Periostin^+^CAFs hyperactivate integrin-FAK/Src axis in lymphatic endothelial cells.**

**A.** Western blot analysis of signaling pathways in HDLECs untreated or treated with CM from NOFs, periostin^-^CAFs and periostin^+^CAFs for 1 h, respectively. **B.** Western blot analysis of the activation of Src and FAK in HDLECs untreated or treated with BMS-354825 monohydrate (Src inhibitor) for 1 h before incubation with CM or with medium alone for 1 h. **C.** Western blot analysis of the activation of Src and FAK in HDLECs untreated or treated with PF-562271 (FAK inhibitor) for 1 h before incubation with CM or with medium alone for 1 h. **D.** Western blot for phospho-Src and phospho-FAK in HDLECs pretreated with blocking antibodies against αvβ3 (2 μg/ml) or αvβ5 (10 μg/ml), and then exposed to CM as indicated.


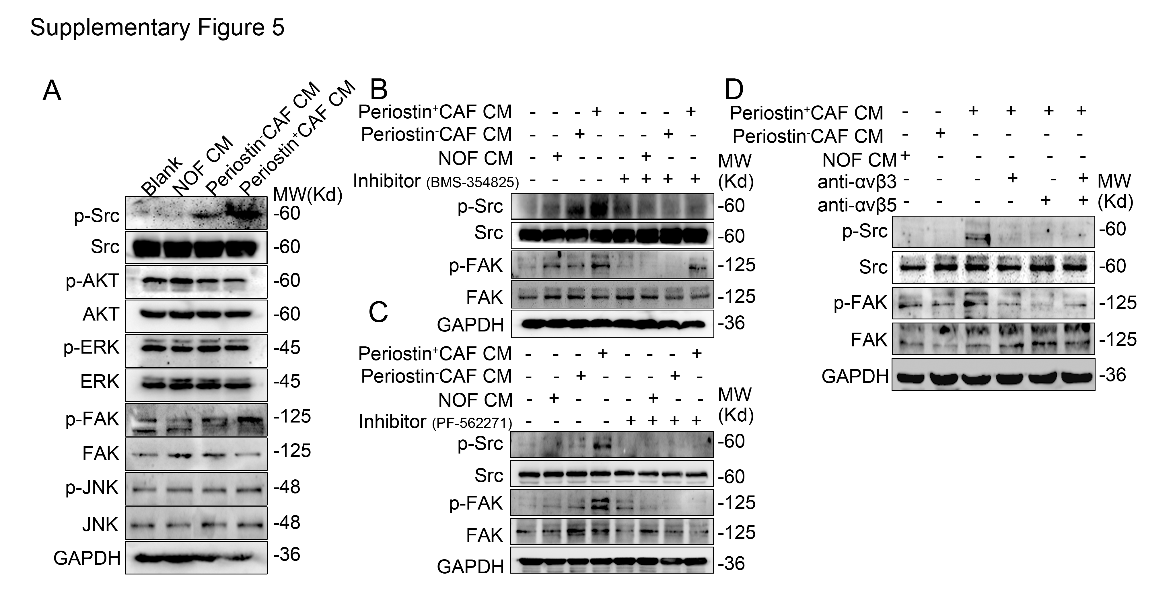


**Supplementary Figure 6. Periostin-induced hyperpermeability is driven by FAK/Src pathway.**

**A-D**. Transendothelial migration assay. **E.** Permeability assay of HDLECs.

**A-B.** HDLECs monolayer following the treatments described in Fig. 5C/5D and the quantification of migrated SiHa-mCherry cells. **C-E.** HDLECs monolayer following the treatments described in Fig. S5A and the quantification of migration of SiHa-mCherry cells. Images are shown at ×200 magnification. Results are representative of three independent experiments in each group. Values are expressed as mean ± SD. * *p* < 0.05.


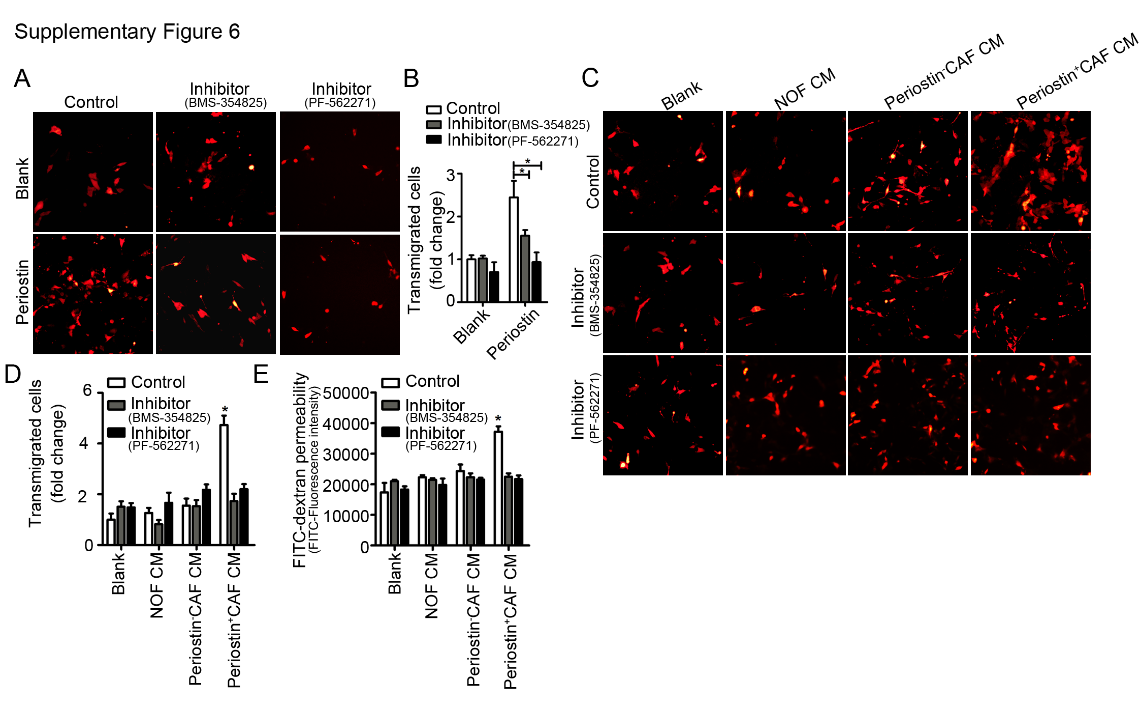


**Supplementary Figure 7. Periostin affects HDLECs permeability by binding to αvβ3 and αvβ5.**

**A-D**. Transendothelial migration assay. **E.** Permeability assay of HDLECs.

**A and C.** HDLECs monolayer following the indicated treatments described in Fig. 5F and quantification of migrated SiHa-mCherry cells. **B, D**, and **E**. HDLECs monolayer following the indicated treatments described in Fig. S5D and quantification of migration of SiHa-mCherry cells. Images are shown at × 200 magnification. Results are representative of three independent experiments in each group. Values are expressed as the mean ± SD*. * p* < 0.05.


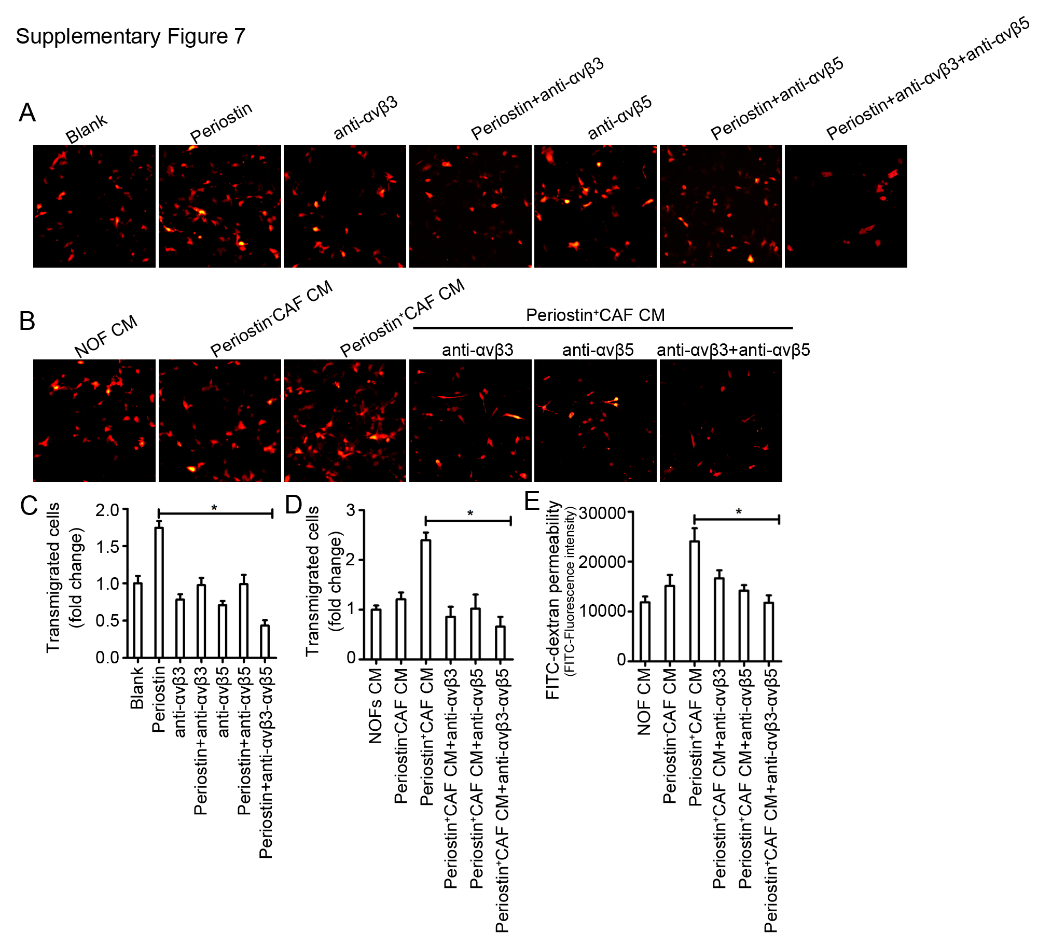


**Supplementary Figure 8. The decreased expression of VE-cadherin induced by periostin were abrogated in FAK/Src inhibitor treated monolayers.**

**A.** HDLECs monolayer treated or not with BMS-354825 Monohydrate/PF-562271 1h were incubated with or without periostin. Endothelial monolayers were imaged by confocal microscopy following immunofluorescence staining of VE-cadherin (red) and nuclei (blue). The decreased expression of VE-cadherin induced by periostin were abrogated in BMS-354825 Monohydrate or PF-562271 treated monolayers. And cell surface mean fluorescence intensity (MFI) was analyzed for VE-cadherin **B**. Images are shown at×400 magnification. Results are representative of 3 independent experiments each in group. Values are expressed as mean ± SD. *. *p* < 0.05.


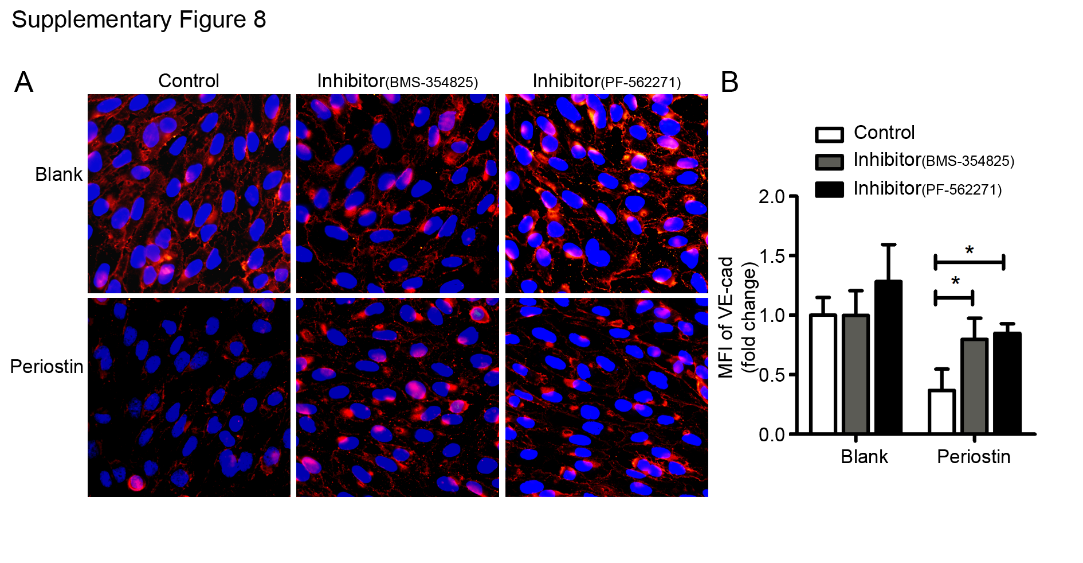


**Supplementary Figure 9. Representative images of CSCC^LNM^ tissues stained for LYVE-1 (green) and VE-cadherin (red) and periostin (purple).**

Low magnification shows that the expression of VE-cadherin in LVs (white arrows) in the periostin-positive region is significantly lower than that (white arrowheads) located further away from this region.


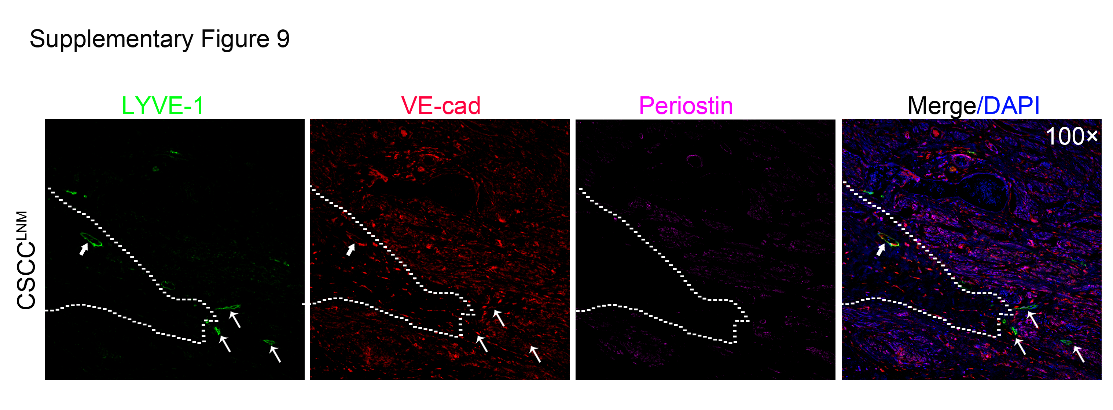


**Supplementary Video 1: Time-lapse view of cancer cell extravasation.**

SiHa-mCherry cells (red) are shown while migrating across the lymphatic endothelial barrier (green). Total movie duration: 24 h, time-step:1 h.


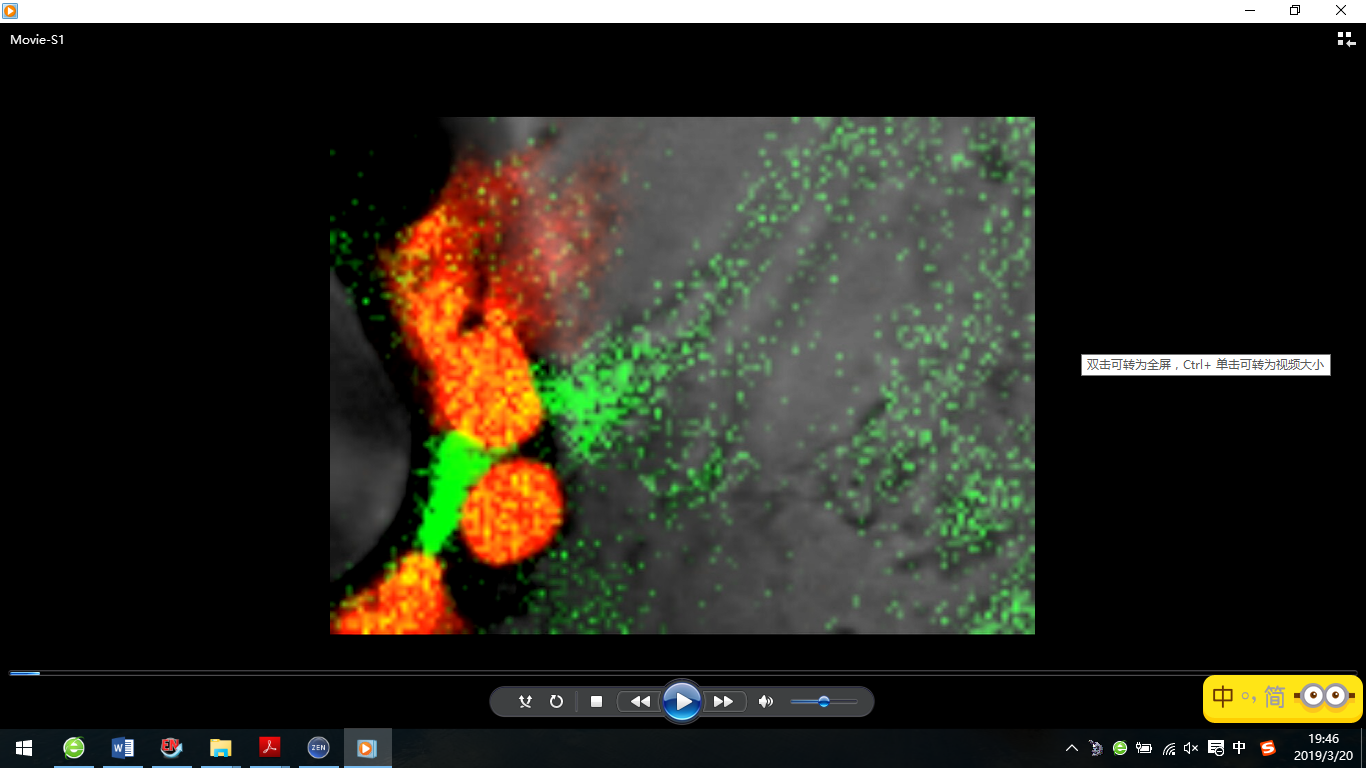

Supplement: Supplementary file 1 — Fig. S1. Quantification of a‐SMA immunofluorescence staining in CSCCnon‐LNM and CSCCLNM samples (p = 0.3448). Fig. S2. Identification of NOFs and CAFs. Fig. S3. The confirmation of Trappin‐2, RGMB, B7H1 expression in all primary fibroblasts by ELISA analysis. Fig. S4. Western blot analysis of ZO‐1, ZO‐2 and Occludin in HDLECs with periostin treatment. Fig. S5. Periostin+CAFs hyperactivate integrin‐FAK/Src axis in lymphatic endothelial cells. Fig. S6. Periostin‐induced hyperpermeability is driven by FAK/Src pathway. Fig. S7. Periostin affects HDLECs permeability by binding to αvβ3 and αvβ5. Fig. S8. The decreased expression of VE‐cadherin induced by periostin were abrogated in FAK/Src inhibitor treated monolayers. Fig. S9. Representative images of CSCCLNM tissues stained for LYVE‐1 (green) and VE‐cadherin (red) and periostin (purple). [file MOL2-15-210-s001.docx]
