# Supplementary material for: Periostin+cancer‐associated fibroblasts promote lymph node metastasis by impairing the lymphatic endothelial barriers in cervical squamous cell carcinoma
Source: Mol Oncol. 2020 Nov 12;15(1):210–27. doi: 10.1002/1878-0261.12837 (PMC7782076; doi:10.1002/1878-0261.12837)
Supplement: Supplementary file 2 — Table S1. Primers for real‐time RT‐PCR. Table S2. Effect of Periostin on popliteal lymph nodes (LNs) metastasis in vivo. [file MOL2-15-210-s002.docx]

Supplementary Table 1. Primers for real-time RT-PCR.

| Gene | PCR primers (5’-3’) | |
| --- | --- | --- |
|  | Sense primers | Antisense primers |
| GAPDH | TGCACCACCAACTGCTTAGC | GGCATGGACTGTGGTCATGAG |
| Periostin | GCTATTCTGACGCCTCAAAACT | AGCCTCATTACTCGGTGCAAA |
| Trappin-2 | CACGGGAGTTCCTGTTAAAGG | TCTTTCAAGCAGCGGTTAGGG |
| B7H1 | TGGCATTTGCTGAACGCATTT | TGCAGCCAGGTCTAATTGTTTT |
| RGM-B | TGTTGGGTATCAGTGACCTCA | CTTCGGGGTTGGTAGAGGATG |

Supplementary Table 2. Effect of Periostin on popliteal lymph nodes (LNs) metastasis in vivo.

|  | NO. total  LNs | NO. positive  LNs | Positive  ratio (%) |
| --- | --- | --- | --- |
| Blank | 10 | 1 | 10% |
| Periostin | 10 | 5 | 50% |
